# Comparison of divided sciatic nerve growth within dermis, venous and nerve graft conduit in rat

**Published:** 2010

**Authors:** Mohammad Javad Fatemi, Kamal Seyed Foroutan, Abass Kazemi Ashtiani, Maryam Jafari Mansoori, Reza Vaghardoost, Sepehr Pedram, Aidin Hosseinpolli, Fatemeh Rajabi, Seyed Jaber Mousavi

**Affiliations:** a Associate Professor of Plastic and Reconstructive Surgery, Hazrat Fatemeh Hospital, Iran University of Medical Sciences, Tehran, Iran; b Plastic and Reconstructive Surgeon, Qom University of Medical Sciences, Qom, Iran; c Assistant Professor of Plastic and Reconstructive Surgery, Ghazvin University of Medical Sciences, Ghazvin, Iran; d Veterinary Surgeon, Department of Clinical Sciences, Veterinary Fatuity, Hazrat Fatemeh Hospital, Iran University of Medical Sciences, Tehran, Iran; e Physician, Hazrat Fatemeh Hospital, Iran University of Medical Sciences, Tehran, Iran; f Assistant Professor of Community Medicine, Tehran University of Medical Sciences, Tehran, Iran; g Resident of Community Medicine, Tehran University of Medical Sciences, Tehran, Iran

**Keywords:** Nerve Injury, Nerve Gap, Nerve Conduit, Dermal Tube, Autogenous Nerve Graft

## Abstract

**BACKGROUND:**

Considering the common origin of skin and peripheral nervous system, a tube of dermal layer of skin hypothetically can be an ideal conduit for nerve reconstruction. An experimental study performed to evaluate the nerve regeneration of efficacy into a dermal tube.

**METHODS:**

Sixty male Wistar rats were used. A 10 mm gap was produced in right sciatic nerves. In group A the autogenous nerve grafts were used to bridge the defects. In group B vein conduit were use to reconstruct the gaps. In group C dermal tube were used to bridge the defects. Morphologic studies were carried out after 3 month.

**RESULTS:**

The density of nerve fibers was significantly higher in autogenous nerve graft group. The efficacy of nerve growth into the dermal tube group was significantly poor in comparison to other groups.

**CONCLUSIONS:**

In the present study, dermis was used as the nerve conduit for the first time. This study indicates that the dermal tube is not a suitable conduit for nerve regeneration till further studies to resolve the problems before clinical application.

Restoration of peripheral nerve injuries with sufficient functional recovery is a significant consideration in reconstructive surgery.^1^,^2^

The clinical outcome is often unsatisfactory and there is rarely a complete return of function.^3^,^4^

Those injuries that produce a gap in the nerve are more difficult for reconstruction.^1^,^3^

The standard technique for reconstruction of a large nerve gap is autogenous nerve graft.^1^–^4^

When a nerve graft bridge a nerve defect the graft functions as a conduit for regenerating axons. It produces a guidelines and biological environment for nerve regeneration.^1^

Limited sources of nerve grafts, mismatches between host and donor nerves, morbidity of donor area and neuroma formation as complications of nerve graft procedure, has stimulated many researchers to evaluate alternative methods for nerve gap reconstruction.^3^–^6^

One possible alternative method to nerve graft is to use nerve conduits with similar efficacy.^5^,^6^

A tube functions as nerve guidance aid for nerve fibers growth and also minimize scar formation and inflammatory cells invasion. Different natural, synthetic absorbable and synthetic non absorbable materials have been used to make a tube.^4^,^7^

A wide range of natural, synthetic non-degradable, and synthetic degradable vein, tendon, fascia, muscle, silicon, poly (glycolic acid), poly (lactic acid), collagen and bioglass extensively used to bridge gap of the nerve.^4^,^6^–^8^

The disadvantages of non-degradable tubes are foreign body response, scar formation, infection and mechanical impigment.^4^,^7^

Recent research has focused on degradable artificial nerve tube, however, resorption of this conduits may result inflammatory reactions and fibrosis around the nerve.^4^,^6^,^9^,^10^

A large variety of substances such as neurotrophic factors or Schwann cells have been used to promote nerve regeneration, especially in larger gaps.^2^,^6^,^8^

Neural crest stem cells that are the precursor cells for peripheral nervous system have the same emberyonical origin with ectodermal layer. Is there any possibility that this common origin enhance nerve regeneration through a tube from this layer?^2^,^4^

The present study examined the efficacy of dermal conduit in bridging a gap of 10 mm by assessing the morphometric parameters of sciatic nerves in rats.

## Methods

### 

#### Animals

Sixty male rats (Wistar) with the weight of 250-300 g were used in this experimental study. Animals were divided into 3 groups according to the types of nerve conduits.

#### Operative Technique

Each rat was anesthetized separately with an intra muscular injection of Ketamin (50 mg/kg) and Xylazin (5 mg/kg). All procedures were performed by the researcher with microscopic magnification during surgery.

**Figure 1:**
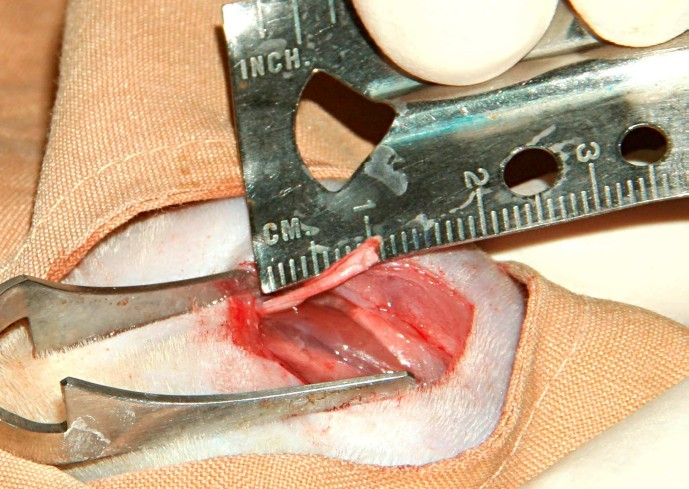
Resection of 10 mm of sciatic nerve.

Using 3 cm incisions to the dorsal gluteal areas and dissection in inter-muscular plan, right sciatic nerves were exposed and the nerve defects were developed by resection of a 10 mm segment of the nerves. An extensive literature search suggests that rat peripheral axons can cross an inter stump gap of 10 mm and it is well documented in a variety of experimental studies (Figure 1).^2^,^5^,^6^

**Figure 2:**
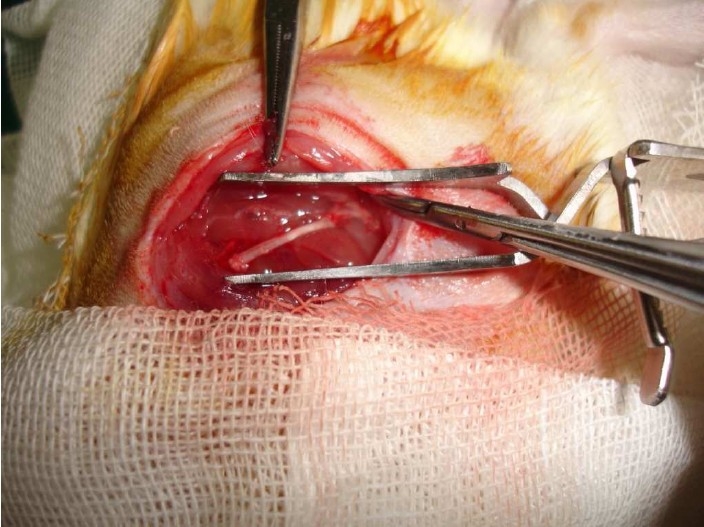
Repair of nerve with interposition nerve graft.

In group A (autogenous nerve graft) (n = 20) the transected segment of the nerve was immediately repaired primarily in a retrograde type (when the nerve is repaired in a retrograde fashion, potential side branching of regenerating axons are prevented). Each nerve end was sutures with four 10-0 nylon (Ethicon) epineural sutures with microscopic magnification and microsurgical instrument (Figure 2).

**Figure 3:**
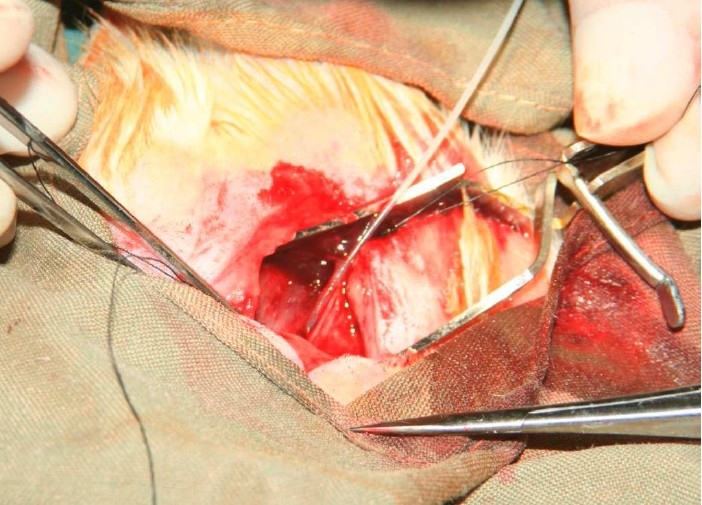
Harvesting and dilating of femoral vein.

In group B (vein tube) (n = 20), a 10 mm segment of femoral vein was harvested through another incision in left inguinal area. The vein was dilated over a no 18 epidural catheter (Figure 3). The vein was grafted in retrograde fashion between the nerve stumps. Both proximal and distal ends were positioned into the vein tube and fixed by four 10-0 nylon (Ethicon) sutures between epineural layer and vein wall.

**Figure 4:**
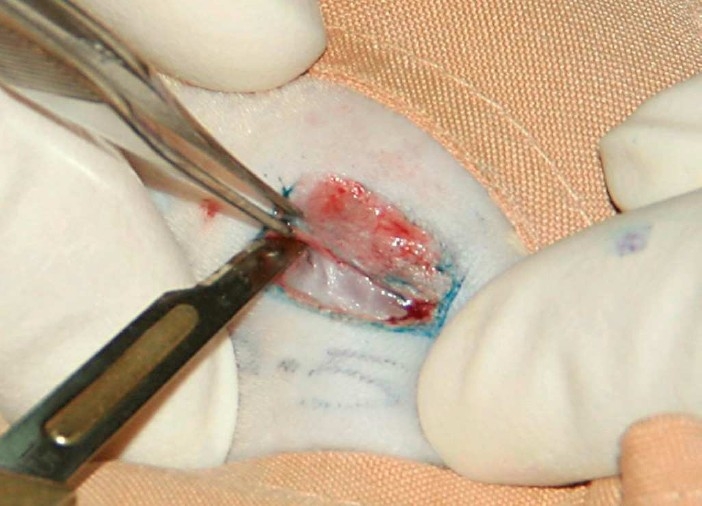
Preparation of dermal lyer.

In group C (dermal tube) (n = 20), the epidermis of a 10×5 mm area from the back of the rat removed (Figure 4). Then a thin dermal layer harvested carefully with loupe magnification and by use of 15 blade surgical knife.

**Figure 5:**
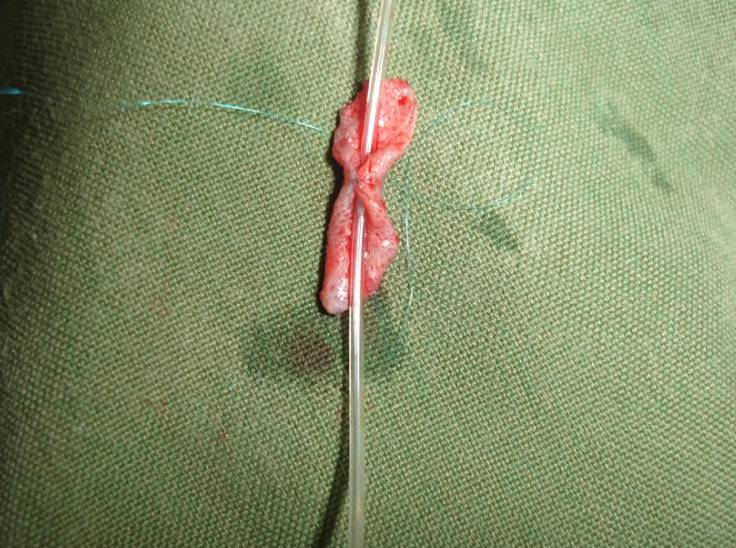
Reconstruction of dermal tube over epidural catheter.

After that a 10 mm dermal tube was made over a no 18 epidural catheter with microscopic magnification and by interrupted 8-0 prolen (Ethicon) sutures (Figure 5). The tube positioned into the nerve gap and after insertion of both nerve ends into the tube each nerve end was sutured. Both proximal and distal part of the nerve were sutured to the tube using four 10-0 Nylon (Ethicon) sutures.

The skin was closed with 3-0 silk interrupted sutures. To prevent infection all animals were received one dose of Enrofloxamin (0.1 mg/kg) before operation and CoAmoxiclave were added to drinking water of rats (1 g/L).

After surgery animals were housed in separate cages whose floors were covered with sawdust. They placed in a standard room with controlled temperature and 12 hour light/dark cycle. Sufficient food and water were provided. After 3 months, rats were killed with an overdose of the anesthetic.

#### Histological Evaluation

Two specimens were cut in each rat. The first specimen was taken from normal sciatic nerve proximal to the conduit and the second sample was taken from the mid-point of the tube. The specimens for tissue evaluation were fixed in 10% formalin (Mojallali) and then processing by a series of graded alcohols (Visian), Xylol (Mojallali) and paraffin (Sacura). Then specimens were embedded in paraffin blocks (tissue-tek, VIP, Sakura). After fixation, the tissue was cut to 4 μm thicknesses by using microtom (Accu-Cut-SRM, Sakura) and was stained with Hemotoxylin and Eosin. All samples were observed under light microscope (OlympusBH _2_) (400 X) and the number of axons in the mid-part of the tubes were compared with the normal proximal sciatic nerves.

#### Statistical Analysis

All the collected data were expressed as mean and standard deviation. Statistical comparisons between there groups were made by SPSS version 16. The ANOVA test was then used as a post hoc test. The meaningful differences was set at p < 0.05.

## Results

All animals tolerated surgical procedures and survived well without any serious surgical complication. During dissection, there was not evidence of infection around the conduits in all groups. In dermal tube group, moderate adhesion and developed fibrotic tissue were observed around the conduit and the nerve. Morphologic analysis that was included density and also average area of the nerve fiber was performed in all groups, comparing them to the normal proximal sciatic nerve samples.

The percentage of axon’s number comparing to normal proximal nerve are shown in table 1.

**Figure 6:**
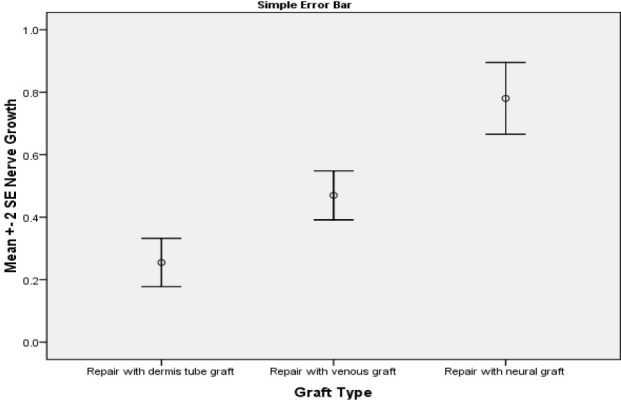
Perecentage of conduit’s axon number to normal proximal nerve

**Table 1:** Distal to proximal nerve growth comparison (mean and standard deviation)

Graft type	Mean	Standard deviation
Autogenous dermal tube	0.225	0.1731
Autogenous vein tube	0.470	0.1750
Autogenous nerve graft	0.780	0.2567

The density of the axon and also average area of the nerve fiber in the middle section of the tube was lower than normal nerve in all rats. In the autogenous nerve graft group, a good regeneration pattern was observed and density and average area of nerve fiber was significantly higher than other groups (p < 0.05).

In the vein tube group, the density and average area of the nerve fiber was significantly higher than the dermal tube group (p < 0.05).

In dermal tube group, fibrous tissues were extended through the entire conduit. Only few scattered axons were observed.

## Discussion

Peripheral nerve has inherent potential for regeneration after trauma because of the ideal microenvironment for growth provided by the distal nerve stump after wallerian degeneration.^6^,^10^,^11^

The standard technique for the reconstruction of a nerve defect is still the use of autogenous nerve graft.^5^

Due to morbidity and limited availability of nerve graft, the use of various nerve conduits following nerve laceration has been initiated clinically.^1^,^12^,^13^

Artificial nerve tubular system containing viable Schwann cells or neurotrophic factors are more promising to bridge peripheral nerve gaps, than using a conduit alone.^9^,^14^,^15^

Wang et al used an inside-out vein tube as interposition graft and stated that it has advantages over other techniques such as nerve graft or standard vein graft. They concluded that adventitia and the medial layer promote nerve regeneration due to rich sources of collagen and Laminin.^3^,^16^

In the present study, dermis was used as the nerve conduit for the first time. Theoretically, the rich source of collagen and the common origin of dermis with peripheral nervous system can promote nerve regeneration.^8^,^17^

Other studies have shown that regenerated axons are able to bridge the nerve tubes implanted between nerve end in rat sciatic nerve if the defect is equal or smaller than 10 mm.^8^,^18^,^19^

Most studies used morphometric analysis in the mid-portion of the conduit to evaluate the regeneration efficacy.^6^,^19^

The growth of axons observed in the dermal tube group indicates that the quality of regenerated nerve was very poor, compared with vein tube and autogenous nerve graft. Also, sever fibrosis, which may be due to collagen, fibroblast or other components of the dermis might be able to prevent axonal growth within this type of conduit. These results suggest that this type of conduit is not a suitable alternative for autogenous nerve graft or vein conduit. The dermal conduit that implanted in this study was well tolerated, but further studies are necessary to test the hypothesis that common origin of skin and peripheral nerves can enhance nerve regeneration in conduits make by this layer.

Besides, the functional recovery was not proportionally improved as the histomorphometric results and further studies are needed to evaluated functional results. Comparison between these types of conduits with artificial tube is another necessary future evaluation. Further more, the addition of nerve growth factors and Schwann cells into the conduit my improve nerve regeneration.^1^

## Conclusions

In conclusion the present study indicates that the regeneration efficiency of dermal tube is significantly lower than autogenous nerve graft and vein tube. It is not a suitable conduit till further studies to resolve these problems before clinical application.

## References

[1] Keskin M, Akbas H, Uysal OA, Canan S, Ayyldz M, Agar E (2004). Enhancement of nerve regeneration and orientation across a gap with a nerve graft within a vein conduit graft: a functional, stereological, and electrophysiological study. Plast Reconstr Surg.

[2] Nie X, Zhang YJ, Tian WD, Jiang M, Dong R, Chen JW (2007). Improvement of peripheral nerve regeneration by a tissue-engineered nerve filled with echtomesenchymal stem cells. Int J Oral Maxillofac Surg.

[3] Eren F, Yuksel F, Ulkur E, Cavdar S, Ercan F, Celikoz B (2005). Nerve regeneration through a healthy nerve trunk: a new and hopeful conduit for bridging nerve defects. Plast Reconstr Surg.

[4] Chen MH, Chen PR, Chen MH, Hsieh ST, Huang JS, Lin FH (2006). An in vivo study of tricalcium phosphate glutaraldehyde crosslinking gelatin conduits in peripheral nerve repair. J Biomed Mater Res B Appl Biomater.

[5] Bunting S, Di Silvio L, Deb S, Hall S (2005). Bioresorbable glass fibres facilitate peripheral nerve regeneration. J Hand Surg Br.

[6] Chiang HY, Chein HF, Shen HH, Yang JD, Chen YH, Chen JH (2005). Reinnervation of muscular targets by nerve regeneration through guidance conduits. J Neuropathol Exp Neurol.

[7] Chang JY, Lin JH, Yao CH, Chen JH, Lai TY, Chen YS (2007). In vivo evaluation of biodegradable EDC/NHS-cross-linked gelatin peripheral nerve guide conduit material. Macromol Biosci.

[8] Lee DY, Choi BH, Park JH, Zhu SJ, Kim BY, Huh JY (2006). Nerve regeneration with the use of a poly (l-lactide-co-glycolic acid)-coated collagen tube filled with collagen gel. J Craniomaxillofac Surg.

[9] Komiyama T, Nakao Y, Toyama Y, Vacanti CA, Vacanti MP, Ignotz RA (2004). Novel technique for peripheral nerve reconstruction in the absence of an artificial conduit. J Neurosci Methods.

[10] Lee YH, Shieh SJ (2008). Secondary nerve reconstruction using vein conduit grafts for neglected digital nerve injuries. Microsurgery.

[11] Tomita K, Kubo T, Matsuda K, Hattori R, Fujiwara T, Yano K (2007). Effect of conduit repair on aberrant motor axon growth within the nerve graft in rats. Microsurgery.

[12] Li J, Yan JG, Ai X, Hu S, Gu YD, Matloub HS (2004). Ultra structural analysis of peripheral-nerve regeneration within a nerve conduit. J Reconstr Microsurg.

[13] Donaldson J, Shi R, Borgens R (2002). Polyethylene glycol rapidly restores physiological functions in damaged sciatic nerves of guinea pigs. Neurosurgery.

[14] Meek MF, Coert JH (2002). Clinical use of nerve conduits in peripheral-nerve repair: review of the literature. J Reconstr Microsurg.

[15] Brunelli GA, Battiston B, Vigasio A, Brunelli G, Marocolo D (1993). Bridging nerve defects with combined skeletal muscle and vein conduits. Microsurgery.

[16] Wang KK, Costas PD, Bryan DJ, Eby PL, Seckel BR (1995). Inside-out vein graft repair compared with nerve grafting for nerve regeneration in rats. Microsurgery.

[17] Colin W, Donoff RB (1984). Nerve regeneration through collagen tubes. J Dent Res.

[18] Evans GR, Brandt K, Katz S, Chauvin P, Otto L, Bogle M (2002). Bioactive poly (L-lactic acid) conduits seeded with Schwann cell for peripheral nerve regeneration. Biomaterials.

[19] Ceballos D, Navarro X, Dubey N, Wendelschafer-Crabb G, Kennedy WR, Tranquillo RT (1999). Magnetically aligned collagen gel filling a collagen nerve guide improves peripheral nerve regeneration. Exp Neurol.

